# An Overview of Arrhythmias in Pregnancy

**DOI:** 10.14797/mdcvj.1325

**Published:** 2024-03-14

**Authors:** Kamala P. Tamirisa, Estefania Oliveros, Shweta Paulraj, Adriana C. Mares, Annabelle Santos Volgman

**Affiliations:** 1 Texas Cardiac Arrhythmia Institute, Dallas, Texas, US; 2 Temple University Hospital, Philadelphia, Pennsylvania, US; 3 SUNY Upstate Medical University, Syracuse, New York, US; 4 Yale University Medical School, New Haven, Connecticut, US; 5 Rush University Medical Center, Chicago, Illinois, US

**Keywords:** antiarrhythmic, maternal arrhythmias, pregnancy, cardio-obstetrics

## Abstract

Cardiovascular disease significantly jeopardizes pregnancies in the United States, impacting 1% to 4% of pregnancies annually. Among complications, cardiac arrhythmias are prevalent, posing concerns for maternal and fetal health. The incidence of arrhythmias during pregnancy is rising, partly due to advances in congenital heart surgery and a growing population of women with structural heart disease. While most arrhythmias are benign, the increasing prevalence of more serious arrhythmias warrants a proactive approach. Guidance and reassurance suffice in many cases, but persistent symptoms require cautious use of antiarrhythmic drugs or other therapies for a safe outcome. Managing more serious arrhythmias requires a comprehensive, multidisciplinary approach involving specialists, including maternal-fetal medicine physicians, cardiologists, electrophysiologists, and anesthesiologists.

## Introduction

Cardiovascular disease significantly jeopardizes pregnancies in the United States, impacting 1% to 4% of pregnancies annually. Palpitations and arrhythmias in pregnancy are common and may lead to concern for the well-being of both the mother and the fetus(es). Pregnancy is associated with a greater risk of arrhythmias due to multiple causes, including neurohormonal and autonomic changes, expanded blood volume, a roughly 20% increase in the heart rate from prepregnancy rates, decreased parasympathetic and increased sympathetic activity, and emotional changes.^1,2^

An increasing trend in maternal mortality has been reported in the United States, and as of 2019, maternal deaths are estimated to be around 20.1 per 100,000 live births.^3^ The risk of arrhythmias is high in women with structural heart disease, and arrhythmias during pregnancy are significant predictors of cardiac events during pregnancy.^4^ Increased maternal age, cardiovascular disease, longevity of congenital heart disease patients, and cardiovascular comorbidities contribute to the increased risk of arrhythmias.^5,6^

## Supraventricular Arrhythmias

### Benign Arrhythmias

Pregnancy is a common (about 8%) inciting event for inappropriate sinus tachycardia with no impact on maternal or fetal outcomes.^7^ Premature atrial contractions (PACs) are common in pregnancy.^8^ Patients with intolerable symptoms from PACs are typically given beta-blockers, chiefly metoprolol, labetalol, and propranolol.^9,10^

### Atrial Tachycardia

Non-atrial fibrillation (AF) supraventricular tachycardias (SVT) are reported in 22 to 33 per 100,000 pregnancies.^11,12^ Approximately 20% of patients with prior SVT have exacerbations during pregnancy.^13^ Although relatively rare in pregnancy, initiating and maintaining atrial tachycardias may be seen.^14^

### Paroxysmal Supraventricular Tachycardia (PSVT)

The most common SVT in pregnancy is atrioventricular nodal re-entrant tachycardia.^8^ Arrhythmias are also more likely in patients with pre-excitation.^15^ Beta-blockers, mainly metoprolol, labetalol, propranolol, and/or digoxin, are the first line for chronic prophylaxis of symptomatic, stable SVT without pre-excitation; verapamil is the second line.^9,10^ In patients with Wolff-Parkinson-White syndrome with symptomatic or frequent SVT, oral flecainide or propafenone is reasonable to prevent further SVT.^10^

In acute-onset SVT, vagal maneuvers are the first-line nonpharmacological treatment. Intravenous adenosine is the first-line pharmacological treatment but may be linked with pre-term labor in the third trimester.^16^ Intravenous beta-blockers (metoprolol, propranolol) are second-line and may be preferred during the third trimester. Atenolol should be avoided due to its association with fetal intrauterine growth restriction.^17^ In refractory cases, non-dihydropyridine calcium channel blockers can be used despite concerns for hypotension, fetal bradycardia, and heart block with verapamil.^18,19^ In limited data, intravenous procainamide has been used safely in pregnancy.^9,20^ In hemodynamically unstable patients, synchronized electric cardioversion with energy dosing similar to a nonpregnant patient is recommended and safe.^10^

Amiodarone is a class D medication with risks for thyroid disorders, bradycardia, and fetal growth restriction. It is reserved for life-threatening circumstances.^18,19^

### Atrial Fibrillation and Atrial Flutter in Pregnancy

Since 2001, atrial fibrillation (AF) has surpassed paroxysmal supraventricular tachycardia (PSVT) as the most frequent arrhythmia seen in pregnant women.^12^ A Registry of Pregnancy and Cardiac Disease (ROPAC) from 2008 to 2011 of 1,321 pregnant women with congenital, ischemic, and valvular heart disease showed a 1.3% incidence of AF or atrial flutter (AFL), with AF occurring mainly in the second trimester. Women with mitral valve disease have a higher incidence. More importantly, compared to women without AF/AFL, maternal mortality was higher in women with AF/AFL (11.8% vs. 0.9%; *P* < .01) and low birth weight (< 2,500 g) (35% vs. 14%; *P* < .02).^21^

In a study of 301,638 pregnant women, the incidence of AF among women with versus without known heart disease was 2.2% versus 0.3%. The incidence of recurrent AF in pregnancy was 39.2% in women with preexisting AF. Among pregnant women with AF, pre-eclampsia and heart failure occurred at a rate of 4.1% and 9.6%, respectively. AF is associated with fetal complications, including premature birth, small for gestational age, intraventricular hemorrhage respiratory distress syndrome, and death.^22^

Pregnant women with new-onset AF should undergo a transthoracic echocardiogram to assess for structural heart disease or new pathology such as pulmonary embolism. Other causes of AF, such as thyroid disease and electrolyte abnormalities, should be assessed and treated.^23^

In patients with hemodynamic compromise with AF, there is a class I recommendation in the 2020 European AF guidelines for immediate direct-current cardioversion,^23^ whereas rate control with beta-blockers and digoxin is recommended for hemodynamically stable patients.^24^ For recurrent or refractory AF, flecainide or sotalol can be used. In general, rhythm control strategies are preferred over heart rate control during pregnancy.^25^

Since pregnancy carries a nearly a 5-fold increased risk of thromboembolic disease, physicians must consider the risk of thromboembolism in pregnant patients with AF. If a pregnant patient experiences AF, electrical cardioversion should be performed within 48 hours to decrease the risk of stroke. Transesophageal echocardiography may be needed if the duration of AF is uncertain. Heparin is preferred, particularly low-weight-molecular heparin, since there is no data on direct oral anticoagulants in pregnant women.^25^ During the first trimester, vitamin K antagonists such as warfarin can be used if the dose is ≤ 5 mg/g, or low molecular weight heparin or intravenous (IV) unfractionated heparin may be used.^26^ Use of low molecular weight heparin should include a periodic evaluation of anti-Xa factor. In order to prevent life-threatening fetal bleeding, women should be converted to IV unfractionated heparin prior to planned delivery.

Non-vitamin K antagonist oral anticoagulants should be avoided due to limited data and experience in pregnancy.^27^

Although data on stroke risk and AF during pregnancy is limited, pregnant women with AF due to mitral stenosis should be on anticoagulants. The CHA_2_DS_2_-VASC score has not been validated during pregnancy, but the 2018 European Society of Cardiology guidelines recommend the same criteria for using anticoagulants as in nonpregnant patients.^25^ The 2023 American AF guidelines recommend shared decision-making since anticoagulation during pregnancy has not been validated in pregnancy.^26^

**Figure 1:**
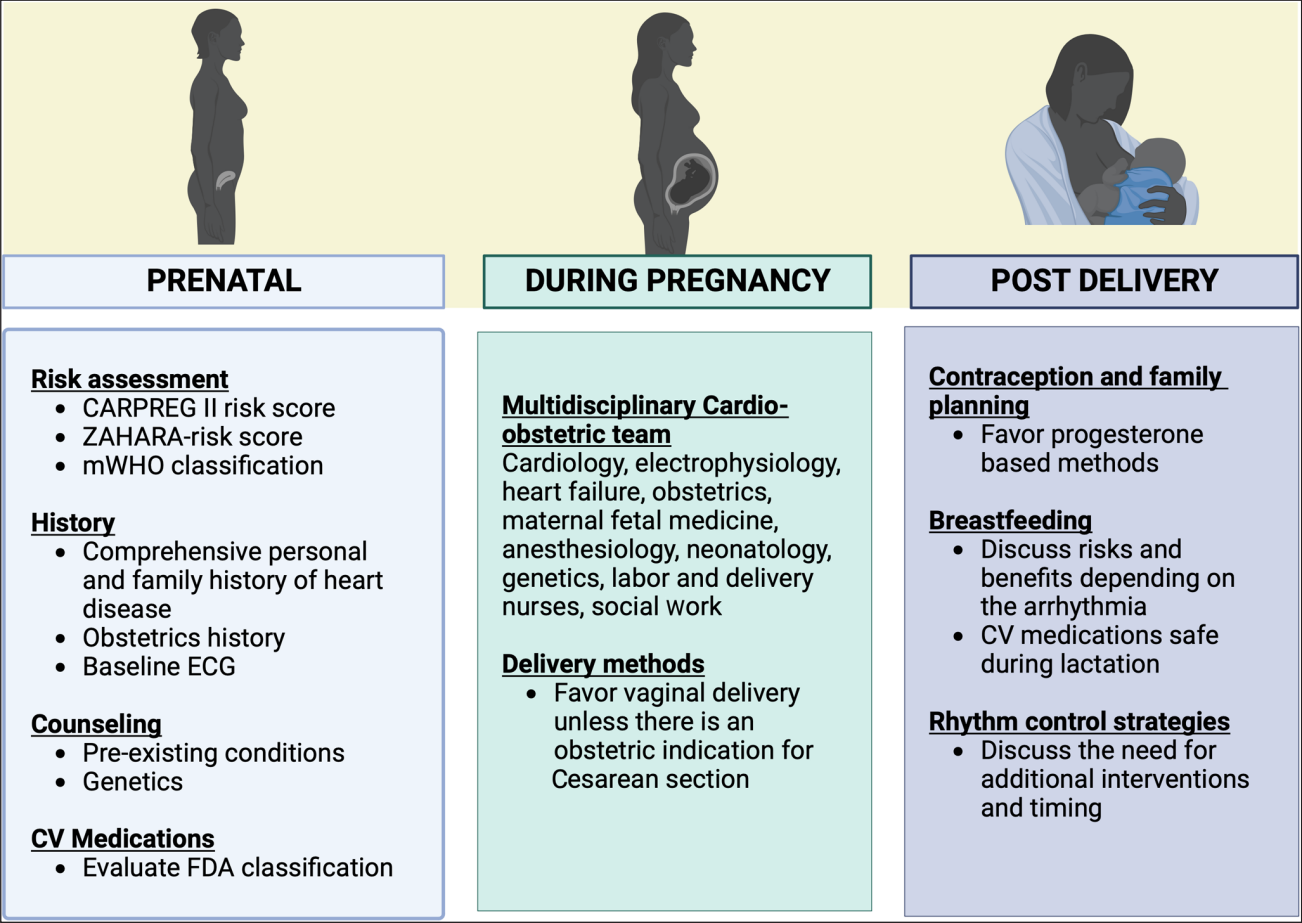
General management of pregnant patients with paroxysmal supraventricular tachycardia and atrial fibrillation. CARPREG: Cardiac Disease in Pregnancy; ZAHARA: Zwangerschap bij Aangeboren Hartafwijking; mWHO: modified World Health Organization: ECG: electrocardiogram; FDA: Food and Drug Administration; CV: cardiovascular.

The increasing incidence of AF during pregnancy underscores the importance of knowing how to evaluate and manage the risk of heart failure and stroke from this arrhythmia. The 2023 American College of Cardiology/American Heart Association/American College of Clinical Pharmacy/Heart Rhythm Society Guideline for the Diagnosis and Management of Atrial Fibrillation is summarized in Table 1, ^26^ and an overview of the management of paroxysmal supraventricular tachycardia and AF is shown in Figure 1.

**Table 1:** Atrial fibrillation recommendations during pregnancy: 2023 Guidelines summary. AF: atrial fibrillation; DCCV: direct current cardioversion; IV: intravenous


AF RECOMMENDATIONS DURING PREGNANCY: 2023 GUIDELINES SUMMARY

**Rhythm Control:** DCCV is safe in pregnancy and should be performed similar to a nonpregnant patient. Fetal monitoring is used during DCCV. In the absence of structural heart disease, pharmacological cardioversion with agents with a history of safe use (IV procainamide) may be used during pregnancy. For maintenance of normal sinus rhythm, agents with a history of safe use (flecainide and sotalol) are reasonable during pregnancy.

**Rate Control:** Rate control can be achieved using agents with a history of safe use (propranolol, metoprolol, digoxin) as first-line agents.

**Anticoagulation: Shared Decision Making is important** Current tools that predict stroke risk in AF are not validated in pregnancy. Most data is extrapolated from managing valvular heart disease patients. First Trimester: Warfarin ≤ 5 mg or low molecular weight heparin or unfractionated heparin Second Trimester: Warfarin or low molecular weight heparin Third Trimester: Warfarin until a week before delivery Switch to unfractionated heparin (or low molecular weight heparin) and stop 4-6 hours pre-delivery


## Ventricular Arrhythmias

### Ventricular Arrhythmias

Ventricular tachycardia (VT) during pregnancy is rare, with a prevalence of 2 per 100,000 hospital admissions; the risk is significantly higher among pregnant women with congenital heart disease, with a prevalence of 4.5 to 15.9 per 1,000 pregnancies.^11,28^ VT occurs at a higher rate in pregnant women with underlying nonischemic cardiomyopathy.^29^ VT in the setting of spontaneous coronary artery dissection and spasm has been reported.^30^ Just like in nonpregnant women, VT in a pregnant patient without structural heart disease is typically hemodynamically well-tolerated.^31^

In pregnant patients with structural heart disease, VT treatment is tailored to the underlying disease. Beta-blockers and lidocaine are safe for hemodynamically stable VT, and Class 1C agents are contraindicated in patients with structural heart disease and coronary artery disease.^32,33^ Conversely, in the absence of structural heart disease, VT is treated with beta-blockers, including propranolol and metoprolol.^34^ Sotalol or flecainide can be used for patients with recurrent and symptomatic VT who are already on beta-blockers. Verapamil is another alternative option for the treatment of fascicular VT.^35^

VT associated with hemodynamic instability should be treated with emergent direct current cardioversion due to the high risk of fetal demise. Higher energies at 100 to 360J, if needed, can be used in life-threatening situations if all other treatments have been exhausted. Even though amiodarone is generally contraindicated, it can be used if all other treatments have failed.^10^ IV magnesium (1-2 mg) can be used for torsades de pointes.^33^

#### Sudden Cardiac Arrest

Maternal cardiac arrest appears to be increasing, occurring in about 1 per 12,000 hospitalizations.^36^ Pregnancy-related hemorrhage and anesthesia complications are the most common causes of sudden cardiac arrest (SCA), but cardiovascular causes can also lead to SCA during pregnancy, especially in cases of advanced maternal age with comorbidities.^37^ Aortic dissection, pulmonary edema, and pulmonary embolism can lead to SCA.^38^ The underlying causes of SCA in pregnancy are often sepsis or hemorrhage, both of which are usually effectively treatable; even so, close monitoring during pregnancy and hormonal changes improve myocardial and cerebral flow during pregnancy. In fact, pregnant women with SCA have been reported to have better outcomes after receiving cardiopulmonary resuscitation (CPR) than nonpregnant women.^39^ Black pregnant patients experiencing SCA have the highest mortality compared with other racial/ethnic groups.^39^

### Inherited Arrhythmia Syndromes

Patients with inherited arrhythmia syndromes (IAS) effectively tolerate pregnancy and lactation. Management includes prepregnancy counseling, disease-specific testing, and optimization of treatment, including medications and defibrillator management. Thorough planning of labor and delivery, review of drugs after delivery, and a newborn cardiology assessment should be part of a multidisciplinary approach.

1. Long QT syndrome is the most common IAS. Risk factors for ventricular arrhythmias include a previous history of VT, SCA, and QTc > 470 msec. The risk of ventricular arrhythmias increases significantly in the 9-month postpartum period, especially for long QT type 2.^40,41^ Long QT syndrome type 1 poses an elevated risk at the time of delivery due to adrenergic stimulation.^42^ Nonselective beta-blockers such as propranolol are the preferred agent, but if the patient is stable on nadolol before pregnancy, it can be continued during the pregnancy.^42^ Mexiletine is the second line of treatment in cases of recurrent VT despite beta-blockers.^42^ Concomitant QTc prolonging therapy should be avoided, and if oxytocin is needed, close telemetry and electrocardiographic monitoring are vital in the multidisciplinary care of these patients.
2. In patients with catecholaminergic polymorphic VT, nonselective beta-blockers or flecainide can be used.^42^
3. Brugada syndrome is much more common in men, and quinidine reduces VT during pregnancy.^43^
4. Pregnancy with a preexisting diagnosis of arrhythmogenic right ventricular cardiomyopathy is well-tolerated, and beta-blockers should not be interrupted. However, pregnancy is contraindicated in patients with biventricular disease and left ventricular ejection fraction of less than 30%.^44^

A summary of the pharmacological treatment options is listed in Table 2.

**Table 2:** Safety of antiarrhythmic drugs during pregnancy and breastfeeding. CNS: central nervous system; FDA: Food and Drug Administration; FGR: fetal growth restriction; FVII: factor VII; IUGR: intrauterine growth restriction; IV: intravenous; SVT: supraventricular tachycardia


DRUG NAME	COMPLICATIONS TO FETAL & NEONATAL	COMPLICATIONS TO PREGNANT WOMEN	FDA RISK CATEGORY	VAUGHAN-WILLIAMS CLASS	TERATOGENIC	USE DURING LACTATION

Adenosine	Consider fetal monitoring, possible small risk of transient fetal bradycardia	Dyspnea Bradycardia Pregnant women may respond to lower doses due to a reduction in adenoside deaminase	C	N/A	No	Safe due to short half-life

Digoxin	Low birth weight Fetal death in toxicity	Miscarriage Monitor maternal levels for toxicity	C	N/A	No	Safe

Lidocaine	Fetal distress may occur in fetal toxicity Bradycardia Acidosis Central nervous system toxicity	–	B	1B	No	Safe

Sotalol	Transient fetal bradycardia Hypoglycemia B-blocker effects Torsades de pointes	–	B	III	No	Safe, but caution advised

Verapamil	Safe (1st choice class IV drug) Trend toward decreased FGR Fetal bradycardia Heart block	Maternal hemodynamic (hypotension) instability if infused rapidly.	C	IV	No	Safe, but caution advised

Flecainide	Concerns over its pro-arrhythmic potential in fetus have limited its use in past	Limited literature for treatment of maternal arrhythmias; however, maternal ingestion used to treat fetal SVT	C	1C	No	Safe

Quinidine	Fetal VIIIn damage Neonatal thrombocytopenia Ototoxicity Premature birth Vestibulocochlear nerve toxicity Torsades de pointe	Rarely, mild uterine contractions	C	1A	No	Safe

Procainamide	Possibly as safe as quinidine short term in pregnancy Drug-induced lupus Torsades de pointes Gastrointestinal disturbance Hypotension Agranulocytosis	–	C	1A	No	Safe for short-term use, but caution advised

Propanolol	Neonatal bradycardia Growth retardation Apnea Small risk of FGR Hypoglycemia	–	C	II	No	Safe

Metoprolol	Neonatal bradycardia Growth retardation Apnea Small risk of FGR Hypoglycemia	–	C	II	No	Safe

Labetalol	–	IUGR Bradycardia Apnea Hypoglycemia Hyperbilirubinemia	Unknown	Unknown	Unknown	Safe

Pindolol	–	–	B	II	No	Safe

Bisoprolol	Bradycardia Hypoglycemia	–	C	Unknown	Unknown	Safe

Disopyramide	–	Premature uterine contractions	C	1A	No	No

Diltiazem	IUGR Fetal death Skeletal abnormalities Fetal bradycardia Heart block Increased risk of FGR	Maternal hypotension	C	IV	Unknown	Safe, but caution advised

Propafenone	–	–	C	1C	No	No

Mexiletine	Bradycardia CNS effects Low Apgar score	–	C	1B	No	Safe, but caution advised

Ibutilide	Torsades de pointes		C	III	Unknown	Safe

Nadolol	Small risk of apnea FGR Hypoglycemia	–	C	II	No	Safe

Atenolol	Greater risk of FGR Cyanosis Low birth weight Growth retardation Neonatal bradycardia Hypoglycemia Other 3 -blockers are preferred over atenolol	–	D	II	No	No

Dofetilide	Human data lacking Torsades de pointes	–	C	III	Unknown	No

Dronedarone	Vascular and limb abnormalities Cleft palate	–	X	III	Yes	No

Amiodarone	Only for short-term use in emergencies. Fetal hypothyroidism Growth retardation Premature birth Goiter Growth retardation Bradycardia Prolonged QT interval IUGR	Bradycardia	D	III	Yes	No

Ivabradine	FGR Bradycardia Hypotension	Bradycardia	N/A	N/A	Yes	No

B-blockers	Avoid atenolol in first trimester due to concern over IUGR Bradycardia Apnea Hypoglycemia Hyperbilirubinemia	–	Unknown	Unknown	Unknown	Safe

Magnesium sulphate	Neuromuscular and/or respiratory depression Skeletal abnormalities	–	D	Unknown	Unknown	Safe

Carvedilol	–	–	C	II	No	Safe, but caution advised

Atropine	Fetal tachycardia Decrease in beat-to-beat variability of the fetal heart rate	Tachycardia	C	Unknown	No	No

Extensive Data/Safe to Use		Moderately Safe/Extensive Experience	Use with Caution in Pregnancy	Do Not Use in Pregnancy		Safety Data Lacking in Pregnancy


## Cardiopulmonary Resuscitation

To avoid aorto-caval compression at or past 20 weeks gestation, manual lateral displacement of the uterus should be considered during CPR. Treatments should not be withheld out of concern for fetal teratogenicity. The protocol for chest compressions, medication doses, and defibrillation energies is similar to that of nonpregnant SCA patients. CPR of the pregnant woman should not be delayed or interrupted for fetal monitoring, although fetal monitoring can be interrupted during CPR. Preparations should be made for perimortem and/or emergency Cesarean delivery.

Pregnant survivors of SCA benefit from a multidisciplinary care team with cardiac electrophysiologists, heart failure specialists, maternal-fetal medicine team, obstetricians, neonatologists, and anesthesiologists.^10^

**Figure 2:**
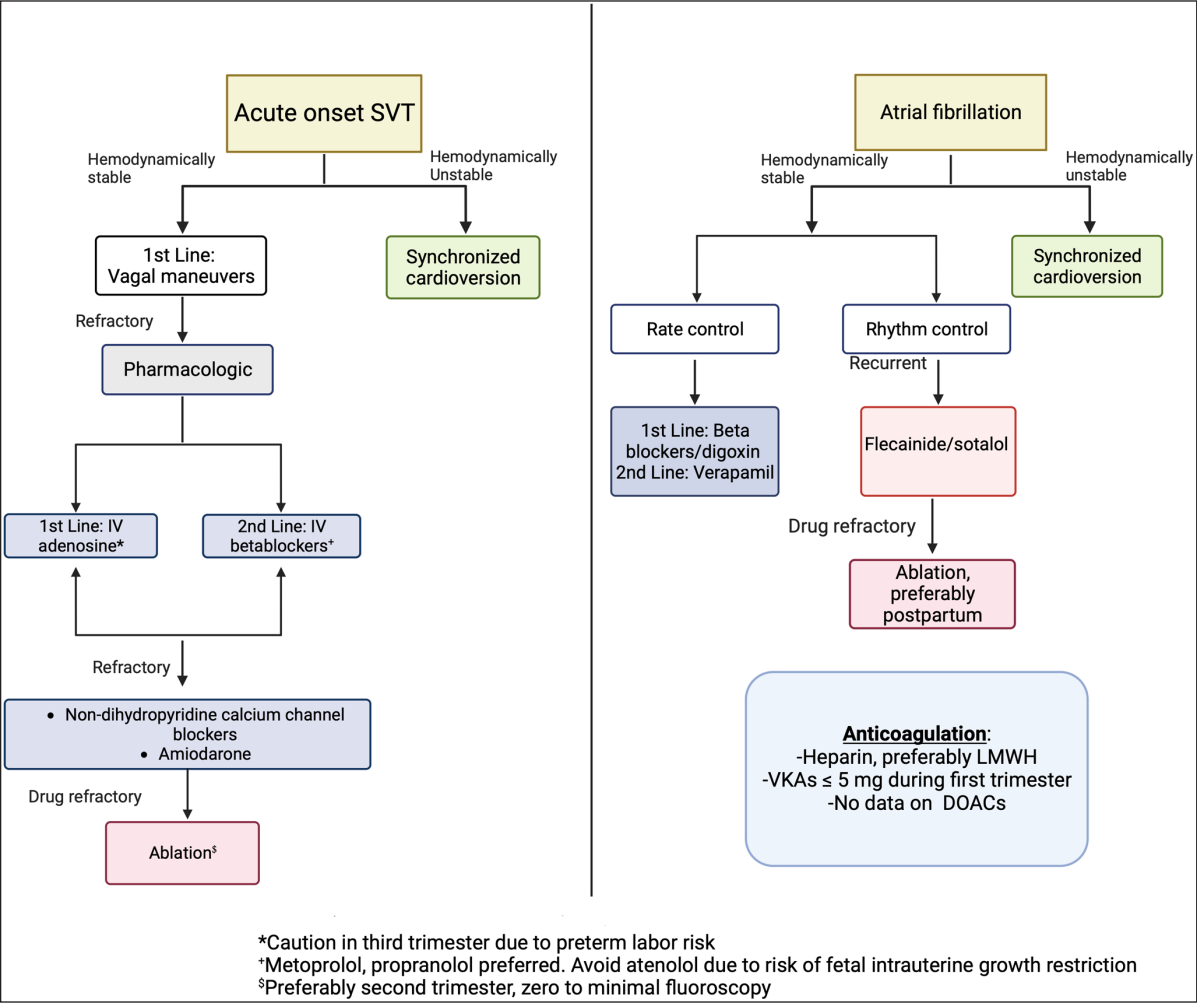
Management of supraventricular tachycardias and atrial fibrillation in pregnancy. LMWH: low molecular weight heparin; SVT: supraventricular tachycardia; IV: intravenous; VKA: vitamin K antagonist; DAOC: direct oral anticoagulant

An overview of management of VT, SCA, and CPR is shown in Figure 2.

## Nonpharmacologic Treatments

### Catheter Ablation

Early electrophysiology consultation for pharmacologic therapy or catheter ablation is recommended in recurrent drug-refractory SVT or tachycardia-induced cardiomyopathy.^44,45^ The radiation dose for common electrophysiology interventions in a fetus is unlikely to exceed the 50 mGy negligible risk threshold for excess malignancy. Abdominal lead shielding leads to a 3% lower radiation dose.^46^ With technological advances, these ablations can be safely performed using nonfluoroscopic electroanatomic mapping, catheter navigation systems, and intracardiac echocardiograms. Catheter ablation should be delayed until the second trimester, if possible.^25,44^ If fluoroscopy is necessary, lead abdominal shielding must be performed.^47^

Catheter ablation may be an alternative option in refractory, symptomatic cases of AF but may need to be deferred after delivery.^25^ Case reports of VT ablation in incessant and recurrent VT have been reported, but ablation is a last-resort option.^48^

### Cardiac Implantable Electronic Devices

#### Pacemakers

Heart rates typically rise by 25% in mid-pregnancy to meet the higher hemodynamic requirements.^49^ Sinus bradycardia is rare and seen in the supine hypotensive syndrome of pregnancy.^25^ Congenital atrioventricular (AV) block may rarely be identified during pregnancy.^50^

Symptomatic complete AV block or heart failure due to chronotropic incompetence may necessitate permanent pacemaker implantation with rate response programming. In the first or second trimesters, implantation can be performed under echocardiographic guidance with electroanatomic mapping, minimizing fluoroscopic use.^51,52^ Permanent pacemaker implantation in pregnancy can be complicated by skin irritation and ulceration at the implantation site due to increasing breast size. A subpectoral pocket may be a suitable implantation site for pregnant women and those planning pregnancies.^53,54^

In patients with symptomatic complete AV block at or near term, temporary pacing followed by early labor induction, if possible, is recommended. Epidural anesthesia to minimize heart rate increase secondary to pain and progression of labor, delivery in the lateral decubitus position to minimize bearing down, or elective instrumental delivery helps shorten the duration of the second stage of labor. The hemodynamic changes quickly revert to prepregnant levels postpartum.^50^ Insertion of a prophylactic temporary pacing lead at the time of delivery is not routinely recommended in cases of stable, asymptomatic AV block with acceptable ventricular rates and narrow QRS.^10^ The presence of previously implanted devices does not increase maternal or fetal risk.^55^

#### Implantable Cardioverter Defibrillator

Patients with risk factors for sudden cardiac death should undergo implantable cardioverter defibrillator (ICD) implantation before pregnancy. Treatment with an ICD during pregnancy increases the risk of major ICD-related complications.^56,57^ Implantation under echocardiographic guidance or electroanatomic mapping can be performed safely, especially if the fetus is > 8 weeks of gestation.^58,59,60^ Shock therapy may need to be disabled, mainly in patients with subcutaneous defibrillators, to minimize inappropriate shocks from over-sensed uterine contractions and myopotentials during labor.^42^

## Special Considerations for the Pregnant Patient

### High-risk Patients

The pillars of cardiac risk assessment in pregnant patients are a comprehensive history and physical examination, 12-lead echocardiogram, and transthoracic echocardiogram.^10^ A personal history of cardiac disease (ie, syncope, congenital heart disease, structural heart disease, channelopathy, acquired heart disease, and isolated cardiac arrhythmias) and family history of sudden death or arrhythmias are as important as the obstetric history. From an arrhythmia perspective, patients with the highest risk are those with the potential for sudden cardiac death or arrhythmias leading to hemodynamic instability affecting maternal-fetal placental circulation. We rely on three models to assess cardiac risk in pregnancy: CARPREG-risk score (CARdiac disease in PREGnancy),^61,62^ ZAHARA-risk score (Zwangerschap bij Aangeboren HARtAfwijkingen I),^63,64^ and the modified World Health Organization (mWHO) classification based on expert consensus.^65^ In the multicenter CARPREG II risk score,^66^ patients with prior cardiac events or arrhythmias have a 15% risk of maternal cardiac complications during pregnancy (ie, cardiac death, cardiac arrest, pulmonary edema, sustained arrhythmia requiring treatment, thromboembolism, stroke, myocardial infarction, and vascular dissection). Most arrhythmias occur in the antenatal period,^66^ which creates a unique opportunity for prepregnancy counseling. In cases of heart failure, the arrhythmias take place in the third trimester or early postpartum.^66^ The ZAHARA risk score relates to congenital heart disease, where a history of prior arrhythmias confers a 7.5% risk of future cardiac complications. If the patient is additionally on cardiac medications, the risk can increase up to 41.5%. In 2011, the European Society of Cardiology guidelines^67^ on managing diseases during pregnancy recommended estimating maternal risk according to the mWHO. From an arrhythmia perspective,^25^ mWHO Class I (very low risk) includes isolated atrial or ventricular ectopic beats; mWHO Class II (low to moderate risk) includes most arrhythmias, mWHO Class II-III is hypertrophic cardiomyopathy and congenital heart disease, and mWHO IV is severe ventricular dysfunction, which puts patients at a high risk for material mortality (and therefore pregnancy is contraindicated). Maternal cardiovascular events occur in 24.2% of cases with higher mWHO classes.^67^ High-risk arrhythmias include both ventricular and atrial arrhythmias. Depending upon the clinical circumstances, both nonsustained and sustained VT can suggest structural heart disease, cardiomyopathies, or cardiac ischemia. In adults with congenital heart disease, atrial arrhythmias are associated with increased risk of mortality.^63^ Pre-pregnancy counseling in patients with Wolff-Parkinson-White syndrome^68^ and congenital long-QT syndrome is warranted.^69^

### Multidisciplinary Cardio-obstetric Team

**Figure 3:**
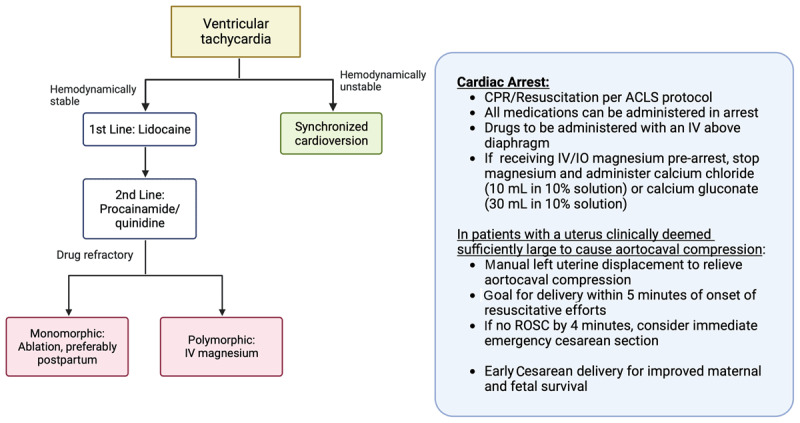
Management of ventricular tachycardia, ventricular fibrillation, and sudden cardiac arrest in pregnancy. CPR: cardiopulmonary resuscitation; ACLS: advanced cardiac life support; IV: intravenous; ROSC: return of spontaneous circulation

Cardio-obstetrics is a new emerging subspecialty that aims to improve maternal and fetal outcomes.^70,71^ The multidisciplinary group has specialists in cardiology, maternal and fetal medicine, obstetrics, anesthesiology, neonatologists, genetics, imaging, labor and delivery nurses, and pharmacy.^72^ The goals of care and management are usually divided into stages: pre-conception, pregnancy, delivery, and postpartum. A multidisciplinary approach including cardiology, electrophysiology, heart failure, obstetrics, maternal-fetal medicine, anesthesiology, and nursing with precise planning and communication of plans for labor and delivery is paramount to ensure the safest possible outcomes for this high-risk medical condition (Figure 3).

### Future Directions

Incorporating machine learning and artificial intelligence into our diagnostic algorithms will continue to evolve and help us identify individuals in the antenatal period who are considered high risk and require additional monitoring. Little data exists on managing arrhythmias during pregnancy and the effect on the pregnant person and the fetus. There is no comparison of effective antiarrhythmic interventions in this patient population. Hopefully, the Registry Of Pregnancy And Cardiac disease (ROPAC) study^73^ and the Randomized Evaluation of Bromocriptine In Myocardial Recovery Therapy (REBIRTH) trial^74^ will continue to provide some insights into pregnant people with cardiovascular disease.

## Conclusions

Palpitations and arrhythmias commonly occur during pregnancy and are usually well tolerated. However, in cases of structural heart disease, arrhythmias can lead to hemodynamic instability with adverse maternal and fetal outcomes. Management of arrhythmias during pregnancy involves thorough knowledge of medications, indications and contraindications, and collaborative management. The key to managing arrhythmias in pregnancy is a multidisciplinary approach and building expertise around cardio-obstetrics programs.

## Key Points

- Palpitations and arrhythmias can occur during pregnancy and are usually well tolerated.
- The most common arrhythmias during pregnancy are generally benign and include sinus arrhythmia, supraventricular tachycardias, and premature atrial contractions.
- A multidisciplinary cardio-obstetrics team approach will allow appropriate management of life-threatening arrhythmias during pregnancy.
